# The relationship between health literacy and patient activation among frequent users of healthcare services: a cross-sectional study

**DOI:** 10.1186/s12875-018-0724-7

**Published:** 2018-03-09

**Authors:** Éva Marjorie Couture, Maud-Christine Chouinard, Martin Fortin, Catherine Hudon

**Affiliations:** 1 Département de médecine de famille et de médecine d’urgence, Université de Sherbrooke, 3001, 12e Avenue Nord, Sherbrooke, Québec J1H 5N4 Canada; 2 Département des sciences de la santé, Université du Québec à Chicoutimi, 555, boulevard de l’Université, Saguenay, Québec G7H 2B1 Canada; 3 Centre de recherche du Centre hospitalier universitaire de Sherbrooke, 3001, 12e Avenue Nord, Sherbrooke, Québec J1H 5N4 Canada

**Keywords:** Frequent users, Primary care, Health literacy, Patient activation, Chronic disease

## Abstract

**Background:**

Frequent users of healthcare services are a vulnerable population that deserves attention due to high costs and negative outcomes such as lower quality of life and higher mortality. Healthcare systems should offer interventions tailored to their needs and to their level of health literacy, including strategies to promote activation. The relationship between health literacy and patient activation remains to be explored. The aim of this study was to examine the association between health literacy and patient activation in a population of frequent users of healthcare services with chronic diseases.

**Methods:**

Cross-sectional data were collected (before randomization) through a clinical trial evaluating a case management intervention in primary care. Participants (*n* = 247) were recruited from the list of frequent users of 4 Family Medicine Groups (FMG) in the Saguenay-Lac-St-Jean region of Québec (Canada). They completed questionnaires by self-report during an encounter with a research assistant: (1) the Newest Vital Sign (NVS) to evaluate health literacy (independent variable); and (2) the Patient Activation Measure-13 (PAM-13) to evaluate patient activation (dependent variable). The relationship between health literacy and activation was examined using biserial correlations.

**Results:**

No association was found between health literacy (independent variable) and patient activation (rb = 0.075, ρ = 0.07) for this population of frequent users of healthcare services.

**Conclusions:**

This study suggests that there is no relationship between health literacy and patient activation among frequent users of healthcare services.

**Trial registration:**

NCT01719991. Registered October 25, 2012.

## Background

It is known that 10% of patients use 80% of healthcare system resources [1, 2]. These frequent users of services present more chronic conditions, a higher level of psychological distress, as well as increased hospital admissions and mortality rates [3–5]. They represent a great challenge for the healthcare system in attempting to optimise care, even more so in a context of chronic disease management. Healthcare systems should offer interventions tailored to their needs [6, 7], including strategies to promote patient activation.

According to Hibbard et al., patient activation is defined as “someone’s knowledge, skills, confidence and behaviors needed for self-managing one’s condition or health^”^ [8]. They defined four (4) levels of activation: 1) believing the patient role is important, 2) having the confidence and knowledge necessary to take action, 3) actually taking action to maintain and improve one’s health, and 4) staying the course even under stress [9]. Although they are related concepts, patient activation is different from patient empowerment, which relates to the process where patients take control of decisions and actions linked to their health [10]. High patient activation levels was shown to be associated with better self-management and healthy behaviours [11, 12], better use of screening services and eventually decreased costs for the healthcare system [12]. Older age, low level of education or poor income was found to be associated with lower levels of activation [8, 9, 11, 12].

Although the operationalization of health literacy is debated, the World Health Organization (WHO) proposes a definition: “the cognitive and social skills which determine the motivation and ability of individuals to gain access to, understand and use information in ways which promote and maintain good health” [13]. The National Adult Literacy Survey (NALS) reported that 75% of patients with chronic diseases had limited literacy skills [14]. A low level of health literacy would be linked to lower treatment compliance [15, 16] and lower preventive care utilization [17, 18]. It was also associated with increased hospital admissions [17, 18], greater risk for poorer health condition [17, 19] and increased mortality [4, 20]. Health literacy was raised as an important variable to consider in the management of chronic disease and as a predictor of health condition [21, 22]. A few studies have reported a positive association between health literacy and patient activation [23–27]. However, many included a sample of patients with higher activation or health literacy levels [23–25] compared to the general population [14]. In addition, no study evaluated this association in a population of frequent users of healthcare services with chronic diseases. This association, if present, could prove to be important for care planning for this vulnerable clientele. It would thus be relevant to integrate interventions targeting low health literacy in order to potentially positively impact patient activation.

Although frequent users of healthcare services represent an additional challenge for the health care system, no study has examined the potential link between health literacy and activation in this population. The aim of this study was to examine the association between health literacy and patient activation among frequent users of healthcare services with chronic disease in primary care settings.

## Methods

### Study design

This cross-sectional study presents the secondary analysis of data collected at baseline (T0) from patients recruited for a larger project, V1SAGES, a randomized controlled trial (RCT) aimed to evaluate the effects of a case management intervention for frequent users of healthcare services with chronic disease on patient psychological distress (primary outcome) and patient activation (secondary outcome) [28]. The intervention was delivered by primary care nurses in Family Medicine Groups (FMG) in Quebec, Canada. A FMG is “a group of family physicians who work in close cooperation with nurses to offer family medicine services to registered individuals” [29]. They provide primary care services and offer extended hours of access through scheduled appointments, walk-in clinics, home care, and telephone access and emergency on-call services. A cross-sectional analysis of data collected at baseline (T0) during the V1SAGES project, on the relationship between health literacy and quality of life was also published [30].

### Participants

We identified frequent users of healthcare services by combining healthcare providers’ judgment with data from a database system, in accordance with current recommendations [31]. The family physicians received a computerized list generated by the MAGIC Chronique software (Médiamed Technologies), of their most frequent users of hospital services (≥ 3 emergency room (ER) visits and/or hospitalizations in the previous year) having at least one of the targeted chronic diseases. They then identified the patients with complex care needs they felt could benefit from the case management intervention (*n* = 323) and suggested additional patients who were frequent users of services in their clinic (*n* = 81). Inclusion criteria were patients between 18 and 80 years of age and having at least one chronic disease (diabetes, cardiovascular disease, respiratory disease, musculoskeletal disease, or chronic pain). Patients with serious cognitive problems were excluded. A nurse then contacted the patients to introduce the project and invite them to participate. If interested, a research assistant contacted each patient to provide further details about the study, assess their eligibility and obtain their consent. Among the patients fitting the inclusion criteria at this stage, 157 were excluded: 113 declined to participate, 36 did not meet the inclusion criteria after further screening, 5 did not respond, 1 passed away and 2 were lost to follow-up. Two hundred and forty-seven participants accepted to participate in the intervention and completed the baseline questionnaire (T0).

The software used for patient identification served as a system to support decision-making for family physicians that did not have access to this kind of information until the time of the study. All participants were asked to read and sign a consent form. The research ethics board of the Centre intégré universitaire de santé et services sociaux (CIUSSS) du Saguenay-Lac-Saint-Jean approved this study.

### Measures

Baseline characteristics of participants were measured at T0. Questionnaires were self-administered with the assistance of a research assistant if needed. If the patient could not travel, the questionnaires were administered at the participant’s home.

Health literacy was assessed using the Newest Vital Sign (NVS) [32], a 6-question survey of a nutritional value label, taking approximately 3 min to complete. Validated in primary care, the NVS has good internal consistency (Cronbach α > 0.76) and has a moderate correlation with the Test of Functional Health Literacy in Adults (TOFHLA) (*r* = 0.59, *p* < 0.001). The receiver operating characteristic (ROC) curve for the prediction of the TOFHLA was 0.88 (CI, 0.84–0.93, *p* < 0,001). A score greater or equal to 4 indicates adequate health literacy [32]. Patient activation was measured using the Patient Activation Measure-13 (PAM), based on the definition provided by Hibbard et al. [9]. This self-administered questionnaire includes 13 statements allowing evaluating 4 levels of activation, as described previously, and providing a global score. It is the short version of the PAM-22 and, in regression analysis, it represented 92% of the variation in the 22-item version estimated activation [9]. Its internal consistency (α = 0.84) and test-retest reliability (Intra class correlation = 0.72) were good allowing for its use in a French- speaking population [33]. The French-language versions of these tools were utilized.

Sociodemographic data (age, gender, education and family income) were obtained. Presence of chronic disease and illness burden were assessed using the validated French-language version of the Disease Burden Morbidity Assessment (DBMA) [34, 35].

### Sample size

Sample size was calculated for the randomized clinical trial. Based on Tabachnick [36], for an α = 0.05 and β = 0.20, the minimum sample size required was approximately 110 participants. Our sample size (*n* = 247) allowed us to obtain adequate statistical power for this cross-sectional study.

### Data analysis

Characteristics of participants were described using means and standard deviations (SD) (continuous variables) or percentages (categorical variables). The association between health literacy (independent dichotomic variable, but with the possibility of being a continuous, variable) and patient activation (continuous dependant variable) was examined using biserial correlation. Literacy was used dichotomously as adequate literacy (NVS score ≥ 4) or low literacy. Patient activation was used as a continuous variable with the global result. PASW Statistics 20 (SPSS Inc.) was used to perform all analyses with a 5% significance level.

## Results

Overall, data from 247 participants were included in the analysis (Table 1). Several of these participants (mean age of 60, SD =13 years, 41.6% male) reported high levels of patient activation (level 3, 34.3%; level 4, 30.8%) but a low health literacy level (NVS < 4, 67.5%). Most of the participants had considerable illness burden (DBMA mean 13.4, SD = 8.5) and a high number of chronic diseases (mean of 6).

**Table 1:** Sample characteristics

Characteristic	Participants *n* = 247
Mean age (SD), years	59.9 (13.3)
Male, n (%)	102 (41.6)
Education, n (%)	
< 8 years	36 (14.6)
8 to 12 years	123 (49.8)
Professional/trade school	11 (4.4)
College	52 (21.1)
University	25 (10.1)
Family income in CAD, n (%) 5 missing	
< 10,000$	24 (9.9)
10,000–29,999$	123 (32.6)
30,000–49,999$	75 (30.9)
≥ 50,000$	64 (26.4)
Illness burden, mean (SD) 1 missing	
DBMA	13.4 (8.5)
Health literacy (NVS), n (%)	
NVS < 4	164 (67.5)
NVS ≥ 4	83 (32.5)
Activation (PAM-13), n (%)	
Level 1	39 (15.8)
Level 2	47 (19.0)
Level 3	85 (34.3)
Level 4	76 (30.8)

*CAD* Canadian dollars, *NVS* newest vital sign, *PAM* patient activation measure, *DBMA* disease burden morbidity assessment, *SD* standard deviation

The biserial correlation analysis did not find an association between health literacy and patient activation (rb: 0.075, *p* = 0.07).

## Discussion

This study examined the relationship between health literacy and patient activation among frequent users of healthcare services with chronic diseases in primary care settings. No association was found.

The results of this study are different from others studies evaluating the association between health literacy and patient activation [23–27]. A cross-sectional study by Sheikh et al. [26] among adult patients seen at the ER demonstrated an association between health literacy and patient activation, but this association was no longer significant when adjusted for age. Similarly, a study by Gwynn et al. [27] reported an association between health literacy and patient activation, but this association was not significant among patients with low comorbidity (0 or 1 comorbidity). In addition, these studies could be influenced by covariables, such as age [23, 26, 27], ethnicity [24] and education [27]. Our study focussed on frequent users of healthcare services with chronic diseases. Their activation and health literacy levels were lower than what was found in previous studies on the subject. This could explain the different results. In light of all these studies, the association between health literacy and patient activation could be present only for certain populations such as older individuals, certain cultural groups or among individuals with high level of activation. More research could examine these hypotheses.

The NVS, the TOFHLA, and the S-TOFHLA are all measurement tools validated for health literacy [32, 37, 38], but this is not an easily assessed concept [39] and there is no consensus on any tool [40]. The NVS presents the advantage of being available in a French-language version and takes few minutes to complete (3 min). The PAM is a well-recognized tool for measuring patient activation [41]. It includes aspects such as knowledge, skills, motivation, and confidence.

This study has strengths and certain limits. It is the first study examining the association between health literacy and patient activation among frequent users of healthcare services, and its statistical power was adequate. However, this study presents the secondary analysis of data collected during the RCT mentioned previously. The choice of measurement tools was thus determined before this analysis. Health literacy remains a concept that is difficult to assess and there is no tool universally accepted as the measurement standard [39]. Using a tool with a broader definition of health literacy, that goes beyond the ability to read and comprehend health information could have yielded different results [42, 43]. In addition, the questionnaires were self-administered which may have induced a social desirability bias. Participants may have answered questions more positively than the reality of their situation. Even though we followed current recommendations for the identification of patients [31], the intervention of family physicians in the selection process may have introduced a selection bias. However, considering our sample was composed of frequent users, we do not think this had a major impact on our analysis.

Although this study did not demonstrate an association, the fact remains that health literacy [17, 18, 40, 44] and patient activation [11, 12] are important concepts for all types of clinicians. Considered separately, health literacy [15, 19] as well as activation [24, 45, 46] have numerous demonstrated impacts on global health. The implementation of interventions for the improvement of patient activation as well as health literacy could be more effective than those focussing only on one of these concepts. Future studies could assess the association between health literacy and patient activation among frequent users in general and not only those presenting chronic diseases. Further research projects could use more than one measure of health literacy simultaneously, for example: the NVS, a validated French-language version of the TOFHLA and/or another measurement tool for health literacy concurrently. A next phase could also detect the effect of time and the intervention on the presence or absence of an association between health literacy and patient activation among frequent users of healthcare services.

## Conclusions

This study did not find an association between health literacy and patient activation in a population of frequent users of healthcare services with chronic diseases in primary care settings. An association between health literacy and patient activation could be present but only for certain populations.

## Abbreviations

CAD: Canadian dollars
CIUSSS: Centre intégré universitaire de santé et services sociaux
DBMA: Disease burden morbidity assessment
ER: Emergency room
FMG: Family medicine group
NALS: National adult literacy survey
NVS: Newest vital sign
PAM: Patient activation measure
RCT: Randomized controlled trial
ROC: Receiver operating characteristic
SD: Standard deviation
TOFHLA: Test of Functional Health Literacy in Adults
WHO: World Health Organization
